# Andean Pseudocereal Flakes with Added Pea Protein Isolate and Banana Flour: Evaluation of Physical–Chemical, Microstructural, and Sensory Properties

**DOI:** 10.3390/foods14040620

**Published:** 2025-02-13

**Authors:** Briggith Leiva-Castro, Liliana Mamani-Benavente, Carlos Elías-Peñafiel, Raúl Comettant-Rabanal, Reynaldo Silva-Paz, Luis Olivera-Montenegro, Perla Paredes-Concepción

**Affiliations:** 1 Grupo de Investigación en Bioprocesos y Conversión de la Biomasa, Carrera de Ingeniería Agroindustrial y Agronegocios, Facultad de Ingeniería, Universidad San Ignacio de Loyola, Lima 15024, Peru; leivathalia03@gmail.com (B.L.-C.); liliana.mamani@usil.pe (L.M.-B.); lolivera@usil.edu.pe (L.O.-M.); 2 Departamento de Tecnología de Alimentos, Facultad de Industrias Alimentarias, Universidad Nacional Agraria La Molina, Av. La Molina s/n Lima 12, Lima 15024, Peru; celiasp@lamolina.edu.pe; 3 Escuela Profesional de Ingeniería Agroindustrial, Grupo de Investigación en Ciencia, Tecnología e Ingeniería de Alimentos y Procesos (CTIAP), Facultad de Ingenierías, Universidad Privada San Juan Bautista, Carretera Panamericana Sur Ex km 300, Ica 11004, Peru; raul.comettant@upsjb.edu.pe; 4 Escuela Profesional de Ingeniería de Industrias Alimentarias, Departamento de Ingeniería, Universidad Nacional de Barranca, Barranca 15169, Peru; rsilva@unab.edu.pe

**Keywords:** quinoa and cañihua extrudate, extrusion cooking, flash profile, free sorting task, nutritious snack, pea protein isolate, scanning electron microscopy

## Abstract

In order to obtain a highly nutritious extrudate, a combination of pseudocereals, vegetable protein, and banana flour, a fruit with high sensory acceptability, was used. The objective of the research was to produce a multi-component extrudate (ME) based on cañihua and quinoa with the addition of pea protein isolate and banana flour. The response variables evaluated were composition, expansion, hydration, colour, and hardness properties, as well as the microscopy and sensory characteristics of the flakes produced. These flakes were compared with three commercial extrudates, commercial quinoa-based extrudate (QE), commercial corn-based extrudate (CE), and commercial wheat-based extrudate (WE), which had similar characteristics. The ME showed a higher protein content compared to commercial extrudates (13.60%), and it had significant amounts of lipids, fibre, and ash. The expansion of the ME was like commercial quinoa but significantly lower than the CE and the WE in terms of expansion (*p* < 0.05). Regarding the absorption and solubility indices of the ME, these indicated that it had lower starch fragmentation compared to the commercial CE and WE. In addition, the instrumental hardness of the ME was higher than the commercial ones due to the complex nature of the product. Through scanning electron microscopy (SEM), it was observed that the ME showed some remaining extrusion-resistant starch granules from quinoa and cañihua with the presence of protein bodies. Finally, the flash profile described the ME as having a pronounced flavour, higher hardness, and lower sweetness, and the free sorting task allowed it to be differentiated from commercial extrudates based on its natural appearance and chocolate flavour.

## 1. Introduction

Extrusion technology makes it possible to produce appetising foods with unique sensory characteristics, which are increasingly valued by consumers. Extrudates produced through this process have a low moisture content, resulting in products with a crispy texture and a longer shelf life [1]. Furthermore, due to the effect of cooking and screw shearing during the extrusion process, the digestibility of starch and protein are improved as a result of starch fragmentation and thermal denaturation [2]. Cereals, such as corn, wheat, rice, and oats, but also pseudocereals, such as quinoa and amaranth, and legumes, such as beans, chickpeas, and peas, are used in the production of expanded extrudates [3]. However, the use of non-traditional and underutilised grains, such as cañihua and other Andean grains, in their whole form, could help reduce the glycaemic index, and their bioactive components are capable of modulating metabolic processes with a positive effect on human health [4,5].

Among the pseudocereals cultivated in the Andean region, quinoa production is notable, with Peru being the world’s leading producer, yielding 51,462.59 tons annually [6]. Quinoa (*Chenopodium quinoa*) is a health-promoting gluten-free pseudocereal with hypoglycaemic properties, containing protein (9.1–15.7%), including albumin (35%) and globulins (37%), along with lower levels of prolamins, fat (5–10%), and carbohydrates (67–74%). In addition, it is rich in minerals, such as iron (8–9 mg/100 g) [7]. However, underutilised gluten-free Andean grains have better nutritional attributes compared to conventional grains, and they are capable of surviving severe climatic events, such as droughts or frosts; therefore, such grains are an alternative for crop biodiversification [8]. In this context, cañihua (*Chenopodium pallidicaule*) is an increasingly popular pseudocereal used in the production of extruded products due to its many nutritional and functional benefits. It is characterised as a good source of protein (13.7–18.8%) with an outstanding essential amino acid profile, including casein, and it is notable for its high lysine content (an amino acid deficient in most cereals); moreover, carbohydrates are the main fraction in the grain (63.6–68%). It is a good source of lipids (5.7–8.8%) and include unsaturated fatty acids and linoleic, oleic, and linolenic acids. It has a high content of dietary fibre (6.1–14.6%), especially the insoluble fraction, and minerals, such as potassium, phosphorus, iron, calcium, and magnesium [9]. It shows remarkable levels of phenolic compounds, especially flavonoids (46.1–144.3 mg/100 g) [10].

To enhance the nutritional value of extrudates, plant-based protein sources, including protein isolates from soybeans, chickpeas, and other legumes, have been used. Pea protein isolate is a great source of iron, and it contains all nine essential amino acids that the human body cannot synthesise. Research indicates that consumption of pea protein can help reduce blood pressure, cholesterol, and cardiovascular disease and strengthen the immune system [11]. Pea protein is also non-GMO and non-allergenic, so it is suitable for people with allergies or food intolerances, and it also promotes prolonged satiety [12]. Blends of legumes and pseudocereal proteins provide a complete, balanced, and improved amino acid profile in extruded products [13]. However, this combination could influence the sensory characteristics, especially the taste of the final product. To reduce this effect, a suitable option is banana flour, which, due to its volatile compounds, could minimise this negative effect on the taste of pseudocereals. On a technological level, its significant starch content would minimise the loss of expansion of the extrudates [14]. In addition, the banana is a hypoallergenic fruit rich in dietary fibre, vitamin C, potassium, and vitamin B6 [15].

There are previous studies reported by Muñoz et al. [16] where they incorporated quinoa into healthy gluten-free extrudates due to its good essential amino acid profile. Only a few recent studies on the production of extruded snacks have been found. One of them is the one developed by Tapia et al. [17], who used cañihua because of its high protein content compared to conventional cereals and traditional pseudocereals, such as quinoa and amaranth. Also, Edima et al. [18] used banana flour to obtain extrudates with swelling capacity and reduced water absorption properties. However, there are no reports yet where pea protein isolate is used in expanded extrudates.

Therefore, the objectives of this research were (1) to obtain a gluten-free multi-component extrudate based on quinoa, cañihua, pea protein isolate, and banana flour and (2) to determine its physicochemical, physical, and microstructural properties and to evaluate its sensory attributes compared to commercial extrudates.

## 2. Materials and Methods

The raw materials used were cañihua and quinoa grains along with banana flour, pea protein isolate, and cocoa powder as a secondary ingredient. A mixture of honey and water in a ratio of 40:60 (w:w) was used to coat the finished product.

The pearled cañihua grains belong to the Sahiua variety, which are dark brown to red in colour, and the white quinoa grains were of the INIA Salcedo variety, which were brought from the Research and Social Projection Programme (PIPS-UNALM) in the department of Puno. Likewise, the pea protein isolate used was Profam PeaTM Protein 580 (Glanbia nutritionals, Salt Lake City, UT, USA), while the banana flour (*Musa paradisiaca*) was of the Inguri variety obtained from the company Agroindustria Orgánica S.A.C., Lima-Peru. Finally, the cocoa powder was of the Winter’s brand, which was purchased from the local market in Lima, Peru.

To obtain the flours from the Andean grains, they were first selected to eliminate impurities or any foreign material present; then they went through a milling process, as the cañihua grains were milled in a hammer mill (MMCDG-500X, Galix Tech, San Pedro de Saño, Peru) through a 0.5 mm mesh opening and the quinoa grains were milled in a hammer mill (MV 15–45 I/C, Fitzpatrick Fitzmill, Delran, NJ, USA), which passed through a 0.3 mm diameter sieve.

### 2.1. Samples

Multi-component gluten-free (ME) extruded flakes were developed from a mixture of Andean pseudocereal flours (cañihua and quinoa), pea protein isolate (ProFamTM 580), and banana flour together with the complementary ingredients, as indicated in Table 1. Percentages of the components were obtained through trial-and-error tests based on the greatest expansion of the extruded product. These ME flakes were compared with three commercial extrudates made from quinoa (QE), corn (CE), and wheat (WE) according to the following criteria: similarity in shape, colour, and chocolate flavour.

### 2.2. Extrusion Process

The extrusion process was realised with a co-rotating twin screw extruder (PQ DRX-50, IMBRAMAQ, Rio de Janeiro, Brazil), which had a seven-zone heating profile of 40, 47, 56, 77, 97, 112, and 120 °C, a screw rotation speed of 800 rpm, a feed rate of 51.3 kg/h, a die exit diameter of 9 mm with a barrel length (BL) of 90 cm, and a cutting speed of 150 rpm. The flour was conditioned to a moisture content of 14% before being fed into the feed hopper for extrusion, cooled to room temperature, and then coated with a honey–water solution. Finally, it underwent a drying process at 75 °C using a tray dryer (ALF 100 GA, FISCHER AGRO, Surquillo, Peru) for 45 min until a moisture content of 5% was reached. The dried extrudates were packed in hermetic trilaminate packaging in order to provide an effective barrier against oxygen, moisture, light, and other contaminants and maintain their physico-chemical and sensory properties.

### 2.3. Physico-Chemical Characterisation of Extruded Products

The physicochemical characterisation was determined using official AOAC methods [19]. Moisture was determined according to AOAC 930.15, fat according to AOAC 920.039, ash according to AOAC 942.05.05, protein according to AOAC 984.13, and crude fibre according to AOAC 962.09. Carbohydrate content was determined based on the difference.

### 2.4. Physical Characterisation

#### 2.4.1. Colour Determination

The CIE (Commission Internationale de L’Eclairage) system was used to determine the colour of the extrudates for the ME and the three commercial extrudates: QE, CE, and WE. A colorimeter (PCE-CSM 10, KOSSODO, Berlin, Germany) was used and calibrated with white reference papers before the measurements. The values of L, a*, and b* were recorded for each sample, where L* indicates the brightness level (0 for black and 100 for white), a* represents the variation between green and red, and b* shows the difference between blue and yellow. Five grams of each sample were used, and three replicates were made for each extrudate. Chroma (C) and hue (H) were additionally determined [20].

#### 2.4.2. Expansion and Conversion Properties of Starch

To determine the Sectional Expansion Index (SEI), the diameters of the extrusions were taken according to the method of Álvarez et al. [21]. A digital Vernier calibrator was used to measure the extruder outlet diameter (Do) and the extrudate diameter (D), and the result was obtained using Equation (1). Bulk density (BD) was determined using the millet seed volume displacement method described by Comettant-Rabanal et al. [22]. It was calculated as the ratio of the sample weight (g) to the displaced volume (cm^3^).

(1) $$SEI={\left(\frac{D}{Do}\right)}^{2}$$

The Water Absorption Index (WAI) and the Water Solubility Index (WSI) were determined using a modified method described by Collantes et al. [23]. In total, 0.5 g of the pulverised and sieved extrudate was mixed with 12 mL of distilled water in a pre-weighed centrifuge tube. The mixture was homogenised using a Vortex intermittently (1 min homogenisation and 4 min rest) for 30 min. At the end of the process, the mixture was centrifuged at 6000 rpm for 40 min. The aqueous phase was placed in an evaporation container and dried in an oven at 105 °C for 5 h to constant weight, and the weight of the dehydrated solids was taken. The hydrated gel was weighed, and the WAI and WSI were calculated using Equations (2) and (3).

(2) $$WAI=\frac{waterabsorbed\;(g)}{drysample\left(1-solublefraction\right)\;(g)}$$

(3) $$WSI=\frac{watersolublematter\;(g)}{drysample\;(g)}$$

#### 2.4.3. Hardness

Hardness was assessed using a Texturometer (TA. HD PLUS, Stable Micro Systems, Godalming, UK) based on penetration with a 45 mm diameter cylindrical probe equipped with a 5 kg load cell. The test conditions set for the test were a pre-test speed of 1.0 mm/s, a test speed of 0.5 mm/s, and a post-test speed of 10.0 mm/s. The hardness resulted from the maximum breaking strength, expressed in Newton (N). The results were the average of eight replicates [24]. This procedure was performed for each extrudate (ME, QE, CE, and WE).

### 2.5. Scanning Electron Microscopy (SEM)

The raw materials comprising the ME as well as the ME, QE, CE, and WE extrudates were analysed through SEM using a scanning electron microscope (Scios 2 DualBeam, Thermo Fisher Scientific, Waltham, MA, USA) to examine the surface of the flours used and to determine the surface of the extrudates in three dimensions. The samples were sputtered with a layer of gold with a thickness of 80 nm and then placed in a microscope. Observations were carried out at an accelerating voltage of 10 kV and 15 kV with a work distance of 17 to 18 mm in order to avoid static charges on the sample surface and to improve the electrical conductivity to achieve high resolution and contrast of the images obtained. The particle size was calculated by measuring the average length of representative particles in the acquired SEM images using Image J software (https://imagej.net/ij/).

### 2.6. Sensorial Characterisation

#### 2.6.1. Flash Profile

The sensory test was carried out in the sensory laboratory of the Faculty of Hotel Management, Tourism and Gastronomy of the San Ignacio de Loyola University, Lima, Peru. The tests were carried out in individual booths, with lighting and ambient temperature control. Each consumer was presented with 10 g of the sample in plastic cups identified with three random numbers. All four samples were presented simultaneously at room temperature (21 ± 2 °C). A total of 72 participants were recruited, aged 18–50 years, who regularly consumed breakfast flakes [25]. The flash profile consists of two stages; however, they can be synthesised into a single session to allow for the participation of a large number of consumers [26].

Consumers received information on the basis and methodology of the flash profile [27,28]. In the first stage, participants described the non-hedonic attributes of their own vocabulary, whenever these were sufficiently discriminative. In addition, participants were asked to rinse their mouths with water at room temperature between samples. In the second stage, consumers ranked the samples according to their intentionality of each of the attributes generated in the first stage (individual list of attributes) from lowest to highest [29,30]. Overall, the total session time ranged from approximately 50 to 60 min for each participant.

#### 2.6.2. Free Sorting Task

For the discriminative test of the free sorting task, the sensory evaluations were carried out in individual temperature-controlled booths (21 °C) under normal white lighting according to the recommendations of the International Organisation for Standardisation 8589 (International Organisation for Standardisation, 2007). For the evaluation, untrained panellists received all samples coded with three random letters simultaneously. Room temperature table water was provided to cleanse their palates and avoid taste fatigue between samples. Participants grouped the samples based on their holistic perceived similarities and differences between the samples using their individual criteria, according to the methodology described by Ares and Varela [29] and Pineau et al. [30]. Participants were free to form as many groups as they considered necessary; however, a sample could only belong to one group [31]. Finally, they could describe by sensory attributes the characteristics of the groups formed. Attributes appearing more than 2 times for at least one product were retained, as described by Pineau et al. [30].

### 2.7. Statistical Data Analysis

The results of the nutritional, hardness, and colour analyses were subjected to analysis of variance (ANOVA) and the LSD–Fisher test for mean comparisons, considering a significance level of 5%. For the sensory data obtained through the flash profile, Generalised Procrustes Analysis (GPA) was used, and for the free sorting task method, multidimensional analysis (MDS) and factor analysis (FAST) were used. The XLSTAT 2023 software (AddinsoftTM) and the R Project version 4.2.3 with RStudio version 4.4.1 with the FactoMineR and SensoMineR packages were used for data processing.

## 3. Results and Discussion

### 3.1. Physico-Chemical Characterisation

The results of the proximate chemical composition are shown in Table 2. The protein content of the ME was 13.6%, which was significantly higher than the commercial extrudates (*p* < 0.05), due to the protein content mainly from the pea isolate. This value was similar to the optimised extrudate with the addition of cañihua obtained by Paucar-Menacho et al. [8]; however, the value of the ME was higher than those of the extrudates obtained by Santacruz Soto [32], where he added cañihua to fortify an extrudate based on traditional cereals. Meanwhile, the lipid content of the ME was lower than the QE (*p* < 0.05) due to the fact that no external fat-based coatings were added to this developed prototype to adhere to the flavourings and sweeteners. While the QE had cocoa paste added, an ingredient that contributed lipids, the CE and the WE showed similar amounts of this component (*p* > 0.05). Regarding crude fibre, the ME had 2.20%, which was lower than the QE but higher than the CE and the WE, due to the fact that the Andean grains used were refined.

The carbohydrate content of the ME was 74.5%, which is similar to that reported by Muñoz-Pabon et al. [16] in a quinoa extrudate. This value was influenced by the addition of banana and cañihua flour, which are rich in starch. This component is very important for the expansion of the flakes during the extrusion cooking process, which produces a brittle, porous, and expanded structure that characterises an extruded product [33].

The CE, QE, and ME extrudates showed higher ash content, with values ranging from 2.08% to 3.14% compared to the WE, which showed a value of 1.48% (*p* < 0.05, Table 2), indicating a higher amount of minerals. The high ash content in the CE product is due to fortification with minerals that are not naturally present in the raw materials used, while the QE and the ME included pseudocereal-based flours in their formulation [34]. Abogunrin and Ujiroghene [35] reported an ash value of 1.54% in an extrudate that used corn and quinoa (90:10) while in a 50:50 ratio, an ash value of 1.81%, which could be attributed to the increase in quinoa flour (pseudocereal) in their formulation.

### 3.2. Physical Characterisation

#### 3.2.1. Colour Parameters

The ME and the WE have higher lightness values (L) of 53.98 and 58.3, indicating that they are lighter in colour compared to the other commercial products evaluated (Table 3). The chromatic parameters a* (red) showed positive values ranging from 5.52 to 7.84, indicating values that place them towards reddish brown tones. The commercial product QE stands out for having the highest value, which is attributed to the inclusion of organic cocoa paste in its composition. In contrast, the ME has the lowest value of 5.52, which indicates a moderate presence of red tones, probably due to the characteristic colour effect of cañihua, as it is present in higher proportion. The WE stood out as having the highest value for the colour parameter b* (yellow), with 14.60, indicating a strong presence of yellow tones. The values of the other extrudates ranged from 9.46 to 11.71, also showing a significant presence of yellow shades. High temperatures and rapid screw rotation significantly decrease the L* value and increase the a* and b* values; this is due to the fact that non-enzymatic browning reactions and pigment destruction intensify with temperature, while shearing allows a fractionation that facilitates the exposure of reactive groups to browning, such as amino groups of proteins [36].

The ME and the CE showed low values of chroma (C), a parameter that reflects colour intensity, with values of 12.36 and 11.86, respectively. While the WE had the highest values, indicating higher colour saturation, this characteristic can be attributed to the inclusion of the artificial colouring caramel IV in its formulation. Concerning hue (H), the ME showed the highest value of 63.47, followed by the WE with 62.22, where this parameter belongs to the region of brown shades. According to Aydın and Özdemir [37], food colour is determined by the ingredients used and their proportions in the formulation. The incorporation of cañihua in the ME contributed positively to the colour, giving it predominantly brown tones with hints of yellow. According to Rolandelli et al. [38], the brown colour in extruded products is associated with non-enzymatic browning reactions, such as the Maillard reaction and caramelisation, which occur during the extrusion process under high temperatures. Thus, the brown hue could reflect the intensity of the heat treatment to which the product was exposed.

#### 3.2.2. Expansion and Conversion Properties of Starch

Table 4 shows the expansion properties of the ME and the commercial extrudates. It was observed that the ME has a lower SEI (2.74) compared to the commercial CE and WE (*p* < 0.05), as these samples contain ingredients, such as starch, and additives, such as calcium carbonate, that enhance their expansion through gasification mechanisms at high temperatures. However, the SEI of the ME was like the QE product (*p* > 0.05), as it was free of gelling additives and starch. Also, these values were lower than the cocoa–corn flour extrudates reported by Ondo and Ryu [39]; however, the bulk density was slightly higher (0.27 g/cm^3^). This is possibly due to the severe processing conditions, such as temperature (>120 °C) and moisture (>17%), which increased starch disruption and promoted sectional expansion, thus producing lower bulk densities of the extrudates [40].

The WAI property indicates the degree of starch modification after the extrusion cooking process and the ability of the starch to interact with water molecules. The WAI of the ME and the commercial extrudates ranged from 1.72 to 2.63, and such values were low compared to those reported by Paucar-Menacho et al. [8] and Ondo and Ryu [39] for Andean grain extrudates and cocoa–corn flour extrudates, respectively. This indicates that the ME’s starch fragmentation was lower, suggesting the presence of intact starch and possible lower starch digestibility [41]. The WSI property is related to the soluble starch fragments generated by the depolymerisation of starch after the extrusion process. WSI values were lower in the ME compared to the CE and the WE samples (*p* < 0.05), indicating that the extrusion process did not cause profound changes in the granular structure of the starches of the pseudocereals (cañihua and quinoa) that composed the ME due to the possible interference of cocoa powder, protein from the pea protein isolate, and fibre from the banana flour. Furthermore, the values of this property were directly related to the SEI, as shown by Collantes et al. [23].

#### 3.2.3. Hardness

The hardness of the extrudate was measured as the maximum force required for deformation of the final product upon compression. The textural properties of the samples are summarised in Table 3. The ME had a higher hardness compared to commercial products. Pérez K [42] concluded that a higher hardness is due to the high protein content and indicates that the incorporation of legumes in the production of extruded products produces a reduction in expansion, an increase in density, and resistance to breakage. This characteristic greatly influenced the hardness of the ME. Furthermore, WSI results obtained in the ME suggest that the multi-component extrudate had a low water solubility due to low starch depolymerisation. This physical property of hydration is related to the high hardness of the ME, making this product ideal for use with liquid products, such as dairy beverages [43].

### 3.3. SEM

**Figure 1:**
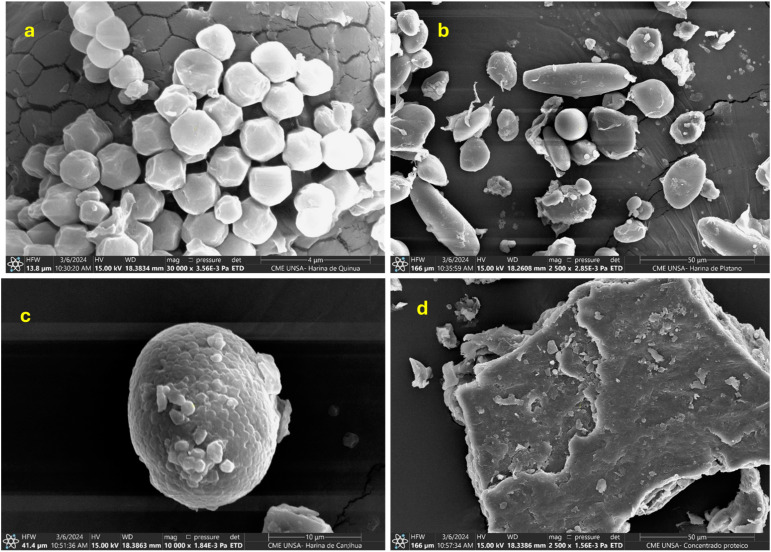
SEM of ME raw materials ((**a**): quinoa flour; (**b**): banana flour; (**c**): cañihua flour; (**d**): pea protein isolate).

Figure 1 shows the morphology of the ME’s raw materials, where in section (a) the partially intact amyloplast and starch granules of the quinoa flour can be seen in the background, forming clusters with a size range between 20 and 40 μm. The surface of the starch granules had a polygonal, semi-spherical, and irregular shape. These quinoa starch granules were approximately 1 to 2 μm in size, in agreement with those reported by Li and Zhu [44] and Romano et al. [45]. In (b), different shapes of the starch granules present in the banana flour are shown, including elongated, oval, elliptical, and compact variants, ranging in size from 10.9 to 36.36 μm in length. Kumar et al. [46] suggested that the substances attached to the structure of these granules are fibres that remained during the milling process. On the other hand, polyhedral and polygonal conglomerate-shaped starch granules were observed in cañihua flour (c). The size of these granules ranged between 0.7 and 1.6 μm, and such dimensions were similar to those found by Pumacahua R. et al. [47], making cañihua starch a nano starch. Moreover, a variety of protein particles ranging in size from 85 to 217 μm were observed in the pea protein isolate (d). These were characterised by compactness and strong attraction behaviour between them. Some of them had a smooth surface and were irregular and rocky in shape. Similar reports have been found in bean protein isolate [48]. The variation in the distribution of starch granules and protein bodies in flour samples can be attributed to the mill’s configuration (clearance, sieve size of the breaking and reduction rollers) [49].

**Figure 2:**
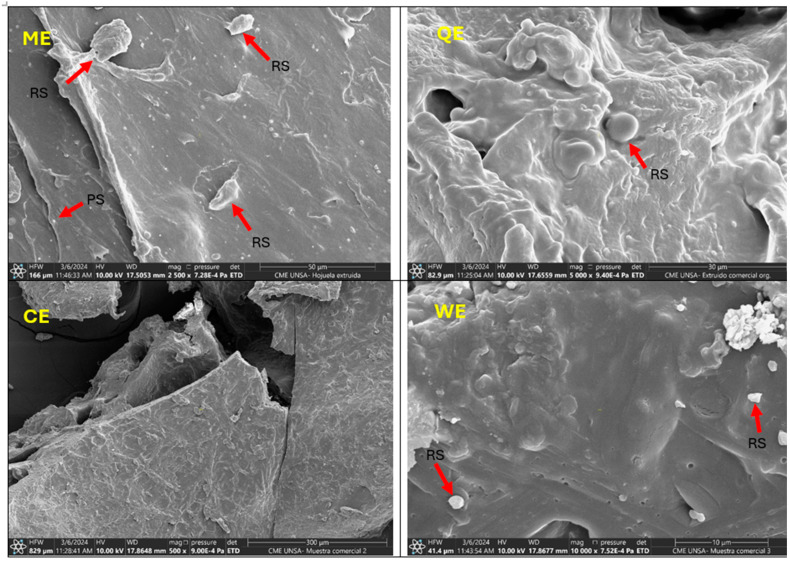
SEM of extruded flakes (ME: multi-component gluten-free extrudate; QE: commercial quinoa-based extrudate; CE: commercial corn-based extrudate; WE: commercial wheat-based extrudate).

Figure 2 illustrates the effects of the extrusion process on the morphology of the extrudates through SEM. All images indicate a porous and heterogeneous structure with irregular air cells. Most of the starch granules were observed to gelatinise during extrusion cooking, creating an expanded volume and a modification of the microstructure, while in others, remnant starch (RS) particles were observed [49]. In the ME, small protein structures (PS) are observed on the surface of gelatinised starch due to pea protein isolate.

The structure of commercial extrudates observed through SEM (Figure 2 QE, CE, and WE) consisted of a dense and compact protein matrix with some remnant starch (Figure 2 QE). Most of the starch granules were melted (Figure 2 CE and WE), with wrinkles on their surface, possibly indicating the presence of fibre from the whole wheat flours they contain. Starch gelatinisation and protein denaturation also occurred, forming a viscoelastic melt, and some dispersed starch granules and air bubbles were observed due to the humid conditions [50].

### 3.4. Sensorial Characterisation

#### 3.4.1. Flash Profile

**Figure 3:**
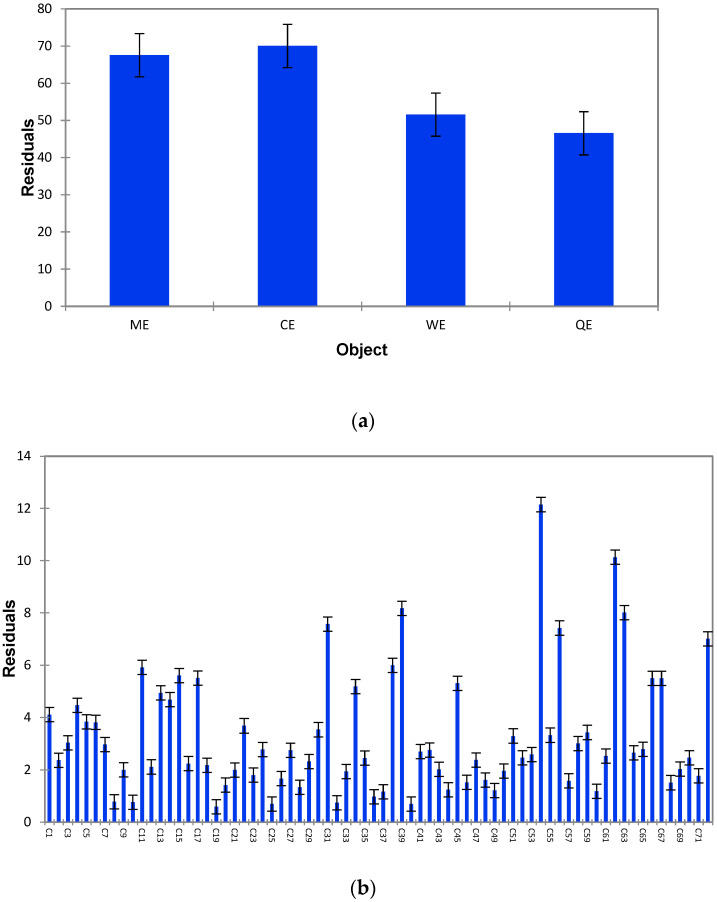
Residues of extruded flakes (**a**) and by configuration (**b**) from the GPA analysis of the flash profile results.

Generalised Procrustes Analysis (GPA) of the flash profile data elucidated the relative positioning of the samples based on the attributes generated by the seventy-two panellists [50]. As illustrated in Figure 3a, the residuals per object (sample) following the GPA indicate that the panellists employed similar criteria in their assessments of the extruded products ME and QE, as well as between the commercial products CE and WE. Notably, however, the QE exhibited the highest reported residual, suggesting that consensus among panellists for this product was significantly lower compared to the others. This discrepancy may imply a greater variability in sensory perceptions or descriptors associated with the QE. In terms of residuals categorised through configuration or by individual panellists (Figure 3b), it was observed that participants C8, C19, C25, C32, and C40 demonstrated a strong agreement regarding the attributes they utilised during evaluation. Conversely, panellists C31, C39, C54, C56, C62, C63, and C72 recorded higher maximum residuals, indicating a lower level of concordance in their sensory evaluations and descriptor generation compared to their peers. This variation highlights the potential impact of individual differences in sensory perception and descriptor selection on the overall consensus achieved through GPA. The findings underscore the importance of considering both individual and collective assessment criteria when interpreting sensory data derived through the flash profile methodology.

**Figure 4:**
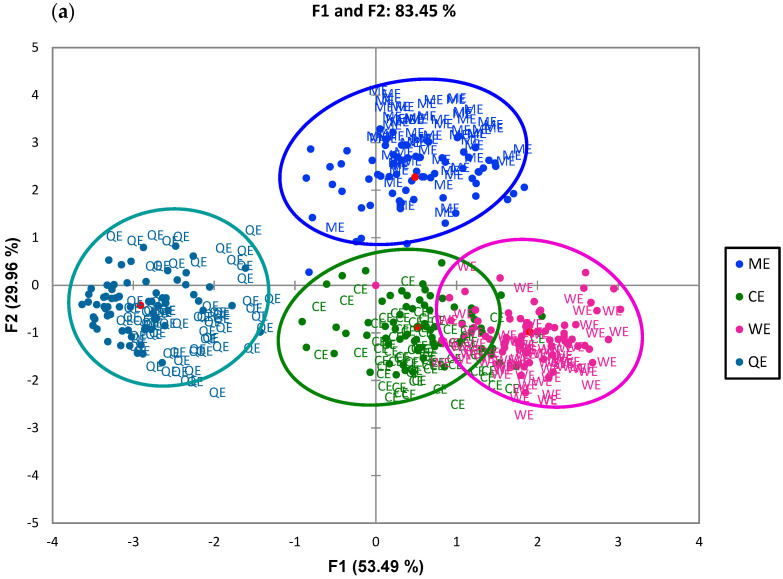

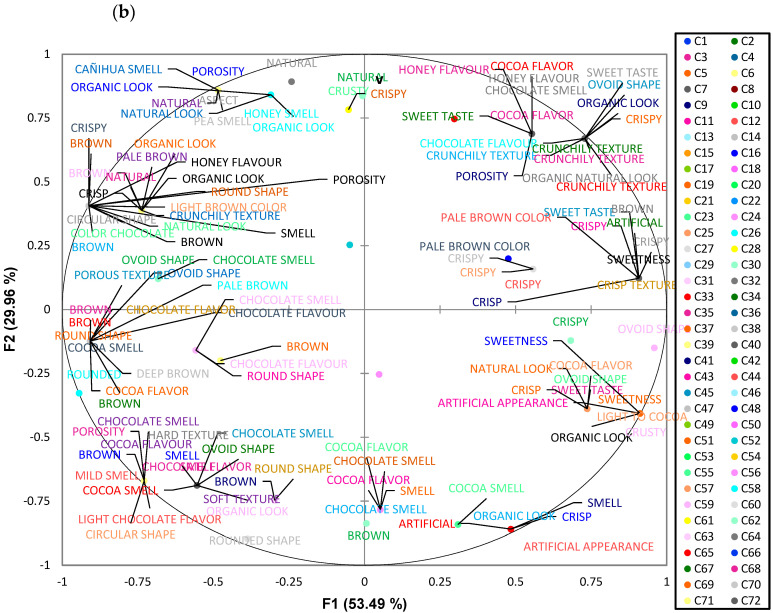
Sensory map based on the GPA of the flash profile results. Consensus for extruded flakes (**a**) and attributes or descriptors of extrudates (**b**).

Figure 4 presents a comprehensive sensory map of the samples alongside the descriptors generated by consumers. In Figure 4a, the first two dimensions derived from the Generalised Procrustes Analysis (GPA) are displayed, effectively illustrating the differentiation among the samples and validating the consensus matrix through an appropriate representation of evaluators’ responses. Notably, these two dimensions account for 83.45% of the total variability in the data, with dimension 1 (F1) and dimension 2 (F2) explaining 53.49% and 29.96% of the variance, respectively. Such high explanatory values are consistent with findings from previous flash profile studies involving gluten-free bakery products and various food matrices, which also reported similar variance percentages exceeding 50% in two-dimensional solutions [50,51,52]. The statistical significance of the distinctions between the extruded products was confirmed with a *p*-value of <0.0001 (alpha = 0.05) in the dimensional test comparing F1 and F2. The analysis reveals that the WE product correlates negatively with both F1 and F2, while the commercial products QE and CE exhibit a positive correlation with F1 and a negative correlation with F2. In contrast, the ME demonstrates a positive correlation with both dimensions. Furthermore, the CE shows a negative correlation across both data dimensions, indicating its distinct sensory profile.

Flavour emerged as a critical attribute for differentiating these four extruded products, as illustrated in Figure 4a. The first dimension, which accounts for a larger portion of variance, is primarily associated with attributes like chocolate flavour and brown colour. Conversely, F2 is more closely linked to sensory characteristics, including shape, sweetness, and crunchiness. Notably, the ME and the WE displayed greater relevance in terms of sensory differentiation compared to other samples, suggesting that these products possess unique characteristics that set them apart. Additionally, a similarity was observed between the commercial corn-based product (CE) and the quinoa-based product (QE). In Figure 4b, it is evident that the WE contains a higher concentration of ingredients compared to the other samples analysed. To date, there are no published studies characterising the sensory profiles of Andean pseudocereal-based extrudates. This study employed the flash profile methodology to effectively identify key sensory attributes, such as sweetness and chocolate flavour, associated with these extrudates while compiling significant sensory descriptors.

#### 3.4.2. Free Sorting Task

**Figure 5:**
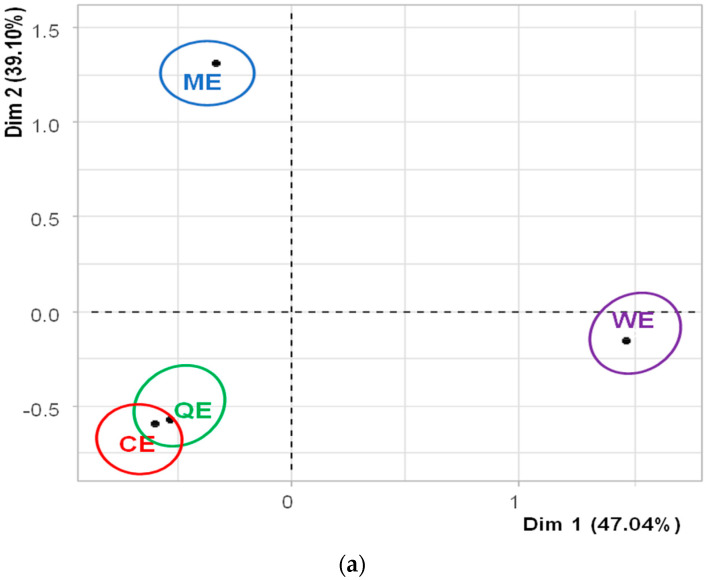

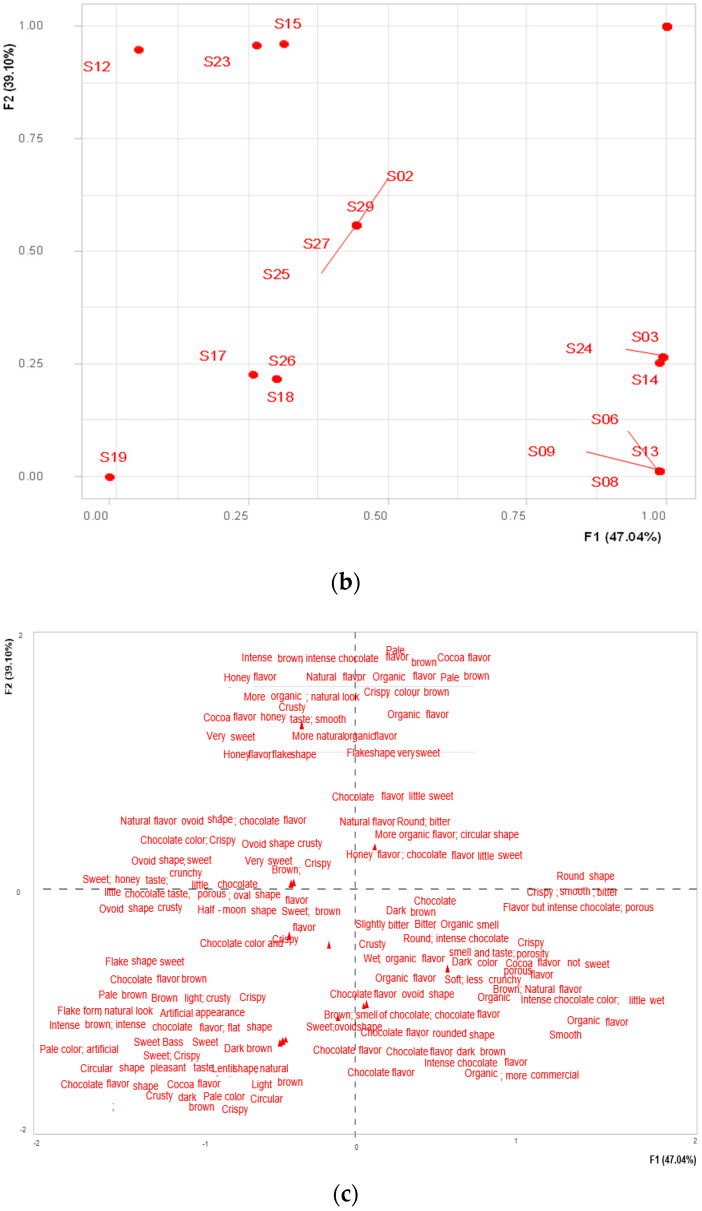
Multivariate analysis for the sorting task method: (**a**) sensory map of the extruded flakes, (**b**) sensory descriptors associated with the samples, and (**c**) representing the consumer based on their sensory perception.

The sorting task method generated a total of 15 different descriptors, which allowed for the generation of six attribute categories: brown colour, rounded shape, chocolate flavour, natural appearance, crunchiness, and sweetness. As shown in Figure 5a,b, the extruded products were grouped into three distinct groups, each characterised by specific sensory attributes. The first group formed by the WE is composed of extruded products made from wheat and distinguished by having the highest concentration of ingredients, including artificial flavours and colours. The inclusion of these additives not only affects the sensory profile of the product but also the consumer’s perception of its quality and authenticity. The second group formed by the ME, representing a gluten-free extruded product, is characterised by descriptors like “natural appearance” and “chocolate flavor”. The ME product is formulated with ingredients from Andean crops, such as quinoa, cañihua, and Peruvian chocolate powder. The differences observed between the WE and the ME can be attributed to variations in the raw materials used and specific parameters of the manufacturing process that impact the final sensory characteristics of the product [53]. Finally, the third group formed the CE and the QE. This group encompasses both commercial extrudates (CEs) and quinoa-based extrudates (QEs), which share similar descriptors, such as “deep brown color” and “sweet”. Despite their similarities, there are few publications documenting significant differences between flakes derived from Andean pseudocereals and those from commercial sources. The observed variations may reflect the heterogeneous responses of consumers during the classification task, as shown in Figure 5c.

The results indicate a wide range of perceptions among consumers regarding the sensory attributes of these extruded products. This variability highlights the subjective nature of sensory evaluation, where individual preferences and previous experiences can significantly influence the selection of descriptors. Furthermore, this diversity in consumer responses could be related to other factors, such as prior knowledge about the ingredients or previous experiences with similar products. Importantly, these findings are consistent with those obtained using the flash profile descriptive method, reinforcing the validity and robustness of the sensory data collected. The convergence between both methods suggests that consumer perceptions are consistent and reliable in sensory evaluation. The sorting task not only allowed for the identification of key descriptors associated with each product; it also underlined the importance of consumer perception in the evaluation of sensory characteristics. These results allow us to know the composition of ingredients and the process parameters that affect the selection based on similarity or dissimilarity as determined by the consumer of the extruded products.

## 4. Conclusions

Four extruded products were analysed, including three commercial products and one new product made with Andean pseudocereals. The new one had the highest protein values compared to the commercial products, and it had a lower lipid value compared to the QE made from quinoa. The colour of the multi-component extrudate showed high lightness (L*) and low a* values, indicating loss of pigmentation, which is associated with the oxidative process caused by the extrusion process. The expansion and water solubility properties of the ME were lower, thus increasing the hardness of this multi-component extrudate compared to the three commercial extrudates. Microscopy analysis revealed the presence of starch remaining after the extrusion process in the multi-component extrudate, possibly indicating lower digestibility of this processed food compared to the commercial ones. According to the flash sensory profiling method, the ME was found to be closely related to the chocolate flavour attribute, brown colour, and crunchiness. In addition, the free ranking task method described the ME as an extruded product with a natural appearance and chocolate taste.

## Figures and Tables

**Table 1:** List of ingredients of the extruded flakes.

Sample	List of Ingredients	Image
ME ^1^	Cañihua flour (59.52%), banana flour (29.76%), pea protein concentrate (5.95%), chocolate powder (3%), quinoa flour (1.77%), and honey and water solution (5%).	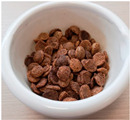
QE ^2^	Organic quinoa, organic cane sugar, cocoa paste, salt.	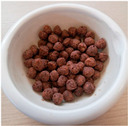
CE ^3^	Cereal grains (whole corn flour, wheat flour, whole wheat flour), sugar, cocoa powder, malt extract, fractionated palm oil, vitamins and minerals, emulsifier (dicalcium phosphate), salt, cocoa liquor, natural flavouring (vanillin), and antioxidant (mixed tocopherol concentrate).	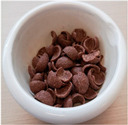
WE ^4^	Fortified wheat flour, sugar, cocoa, marshmallows, malt extract, malt flour, calcium and phosphorus (tricalcium phosphate), salt, glucose, flavourings (chocolate and evaporated milk flavour), iron and vitamins, colouring (caramel IV), emulsifier (sunflower lecithin), sweetener (steviol glycosides from stevia), and antioxidant (concentrated tocopherol blend).	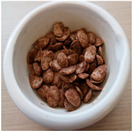

^1^ Multi-component gluten-free extrudate; ^2^ commercial quinoa-based extrudate; ^3^ commercial corn-based extrudate; ^4^ commercial wheat-based extrudate.

**Table 2:** Proximal chemical composition of extruded flakes.

Sample (g/100 g) (d.b.)	Protein (Nt × 6.25)	Lipids	Crude Fibre	Carbohydrates	Ashes	Moisture
ME ^1^	13.60 ± 0.01 ^a^	2.60 ± 0.01 ^b^	2.20± 0.01 ^a^	74.50± 0.07 ^c^	2.08± 0.11 ^b^	5.02 ± 0.02 ^b^
QE ^2^	12.57 ± 0.01 ^b^	14.19 ± 0.03 ^a^	2.56± 0.30 ^a^	61.33± 0.26 ^d^	2.18± 0.06 ^b^	7.16 ± 0.03 ^a^
CE ^3^	9.03 ± 0.07 ^d^	1.01 ± 0.08 ^c^	1.07± 0.03 ^b^	81.12± 0.76 ^b^	3.14± 0.01 ^a^	4.14 ± 0.08 ^c^
WE ^4^	10.06 ± 0.14 ^c^	0.37 ± 0.17 ^d^	0.67± 0.23 ^b^	83.65± 0.13 ^a^	1.48± 0.04 ^c^	3.77 ± 0.03 ^d^

Values represented by mean ± standard deviation (n = 3). ^1^ Multi-component gluten-free extrudate; ^2^ commercial quinoa-based extrudate; ^3^ commercial corn-based extrudate; ^4^ commercial wheat-based extrudate. The different letters in the columns indicate statistically significant differences using the LSD–Fisher test (*p* < 0.05).

**Table 3:** Colour and hardness properties of extruded flakes.

Parameters	ME	QE	CE	WE
L	53.98 ± 0.88 ^b^	49.56 ± 0.39 ^c^	49.60 ± 1.13 ^c^	58.62 ± 0.310 ^a^
a*	5.52 ± 0.19 ^c^	7.84 ± 0.26 ^a^	7.16 ± 0.07 ^b^	7.69 ± 0.26 ^a^
b*	11.06 ± 0.53 ^b^	11.71 ± 0.62 ^b^	9.46 ± 0.44 ^c^	14.60 ± 0.60 ^a^
C	12.36 ± 0.54 ^c^	14.10 ± 0.66 ^b^	11.86 ± 0.39 ^c^	16.51 ± 0.65 ^a^
H	63.47 ± 0.67 ^a^	56.18 ± 0.53 ^b^	52.87 ± 1.02 ^c^	62.22 ± 0.20 ^a^
ΔE*	-	5.77 ± 0.73 ^a^	3.30 ± 0.98 ^b^	5.64 ± 0.41 ^a^
Hardness (N)	46.62 ± 0.01 ^a^	21.91 ± 0.01 ^b^	17.84 ± 0.17 ^c^	13.30 ± 0.09 ^d^

Values represented by mean ± standard deviation (n = 3 for colour and n = 8 for hardness). The different letters in the rows indicate statistically significant differences using the LSD–Fisher test (*p* < 0.05).

**Table 4:** Expansion and starch conversion properties of extruded flakes.

Property	ME	QE	CE	WE
SEI ^1^	2.74 ± 0.13 ^a^	2.58 ± 0.13 ^a^	4.40 ± 0.31 ^b^	4.78 ± 0.38 ^c^
BD ^2^ (g/cm^3^)	0.27 ± 0.01 ^c^	0.26 ± 0.01 ^bc^	0.25 ± 0.01 ^a^	0.25 ± 0.01 ^b^
WAI ^3^	4.46 ± 0.07 ^c^	4.23 ± 0.06 ^b^	2.88 ± 0.13 ^a^	4.23 ± 0.14 ^b^
WSI ^4^	21.42 ± 0.60 ^a^	22.73 ± 1.46 ^a^	51.86 ± 3.03 ^c^	33.15 ± 0.74 ^b^
Extrudate images	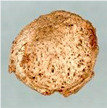	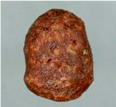	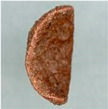	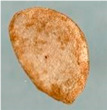

Values represented by mean ± standard deviation (^1^ n = 8 for SEI, sectional expanded index, and ^2^ BD, bulk density; ^3^ n = 5 for WAI, Water Absorption Index, and ^4^ WSI, Water Solubility Index). The superscript letters in the same row indicate statistical differences using the LSD–Fisher test (*p* < 0.05).

## Data Availability

The original contributions presented in this study are included in the article; further inquiries can be directed to the corresponding author.
